# Up modulation of dose‐averaged linear energy transfer by simultaneous integrated boost in carbon‐ion radiotherapy for pancreatic carcinoma

**DOI:** 10.1002/acm2.14279

**Published:** 2024-01-23

**Authors:** Weiwei Wang, Jiayao Sun, Jingfang Zhao, Jingyi Cheng, Guo‐Liang Jiang, Zheng Wang

**Affiliations:** ^1^ Department of Medical Physics Shanghai Proton and Heavy Ion Center Fudan University Cancer Hospital Shanghai Key Laboratory of Radiation Oncology (20dz2261000) Shanghai Engineering Research Center of Proton and Heavy Ion Radiation Therapy Shanghai China; ^2^ Institute of Modern Physics Applied Ion Beam Physics Laboratory Fudan University Shanghai China; ^3^ Department of Radiation Oncology Fudan University Cancer Center Shanghai China; ^4^ Department of Nuclear Medicine Shanghai Proton and Heavy Ion Center Fudan University Cancer Hospital Shanghai Key Laboratory of Radiation Oncology (20dz2261000) Shanghai Engineering Research Center of Proton and Heavy Ion Radiation Therapy Shanghai China; ^5^ Department of Radiation Oncology Shanghai Proton and Heavy Ion Center Fudan University Cancer Hospital Shanghai Key Laboratory of Radiation Oncology (20dz2261000) Shanghai Engineering Research Center of Proton and Heavy Ion Radiation Therapy Shanghai China

**Keywords:** carbon‐ion radiotherapy, dose‐averaged LET modulation, pancreatic cancer

## Abstract

**Background:**

Local recurrence in locally advanced pancreatic cancer (LAPC) after carbon‐ion radiotherapy (CIRT) may partly attribute to low dose‐averaged linear energy transfer (LET_d_), despite high CIRT dose.

**Purpose:**

This study aimed to investigate the approaches to up‐modulate the CIRT LET_d_ and to evaluate the corresponding oxygen enhancement ratio (OER) reduction.

**Methods:**

10 LAPCs that had been irradiated by CIRT with 67.5 Gy (RBE) in 15 fractions were selected. Their original plans were taken as the control plan for the LET_d_ and OER investigations. Our considerations for up‐modulating LET_d_ were: (1) to deliver high doses to gross tumor volume core (GTVcore), while keeping dose constraints of the gastrointestinal (GI) tract in tolerance; (2) to put more Bragg‐peak (BP) within the modulated targets; (3) to increase the BP density, high doses were necessary; (4) CIRT LET_d_ could be effectively increased to small volumes; and (5) simultaneous integrated boost technique (SIB) could achieve the aforementioned tasks. The LET_d_ and the corresponding OER distributions of each type of SIB plan were evaluated.

**Results:**

We delivered up to 100 Gy (RBE) to GTVcore using SIB. The mean LET_d_ of GTV increased significantly by 21.3% from 47.8 to 58.0 keV/μm (*p* < 0.05). Meanwhile, the mean OER of GTVcore decreased by 6.6%, from 1.51 to 1.41 (*p* < 0.05). The GI LET_d_S in all modulated plans were not more than those in the original plans.

**Conclusions:**

SIB could effectively increase CIRT LET_d_ to LAPC, thus producing reduced OER, which may effectively overcome the radioresistance of LAPCs.

## 1 INTRODUCTION

Locally advanced pancreatic carcinoma (LAPC) is a deadly disease. Although chemo‐radiation is proposed as one of the treatment choices, the prognosis is dismal with a median survival time (MST) of 12 to 15 months.^1^ The main reason for the poor outcome is the tumor hypoxia based on intraoperative measurements of tumor oxygenation.^2^ Besides, delivery of high doses to the target is difficult due to the poor irradiation tolerance of the adjacent gastrointestinal (GI) tracts. Finally, the high distant metastasis rate attributes to the poor prognosis.

For LAPC, the local control of the primary tumor is valuable, which could prolong survival and improve patients’ quality of life. In the past decades, carbon‐ion radiotherapy (CIRT) has been used to treat LAPC and yielded an encouraging outcome. The MST was greatly prolonged to over 24 months as reported by Japanese studies.^3^ We believe that the both physical and biological advantages of CIRT contributed to its success. However, there are some rooms to be explored in CIRT to further improve the outcome of LAPC.

The heavy‐ion irradiation experiments showed that the high dose‐averaged linear energy transfer (LET_d_) beams produced a higher fraction of direct action, compared to low LET_d_ beams.^4^ The cell survival curves from sarcoma cell lines showed larger α values after CIRT than x‐ray and proton, which implied a high fraction of direct action.^5^ Thus, high LET_d_ produced a high percentage of direct action. It was known that the direct action damaged DNA with a double‐strand break (DSB). DSB was difficult to repaire. Thus, CIRT killed tumors more effectively than photon radiotherapy with relative biological effectiveness (RBE) of 2 to 3. Moreover, it was found that higher beam LET was associated with higher RBE.^6^ Recently, Monlinelli et al. reported a clinical study of CIRT on sacral chordomas that showed local recurrence was due to the low beam LET, despite of high CIRT dose delivered.^7^ Their experience indicated that the carbon‐ion LET_d_ was also important for tumor control besides the RBE‐weighted dose. Radiation needs oxygen to fix the damage to DNA. The lack of oxygen attenuates this effect. The oxygen enhancement ratio (OER) is used to quantify the level of tumor hypoxia. OER value of 2 means the tumor in hypoxia condition needs to double the radiation dose based on the dose needed in the aerobic condition. Due to the limitations of the organ at risk (OAR) constraints, the treatment outcome of this tumor may be dismal. However, the damaging consequence of the direct action is less dependent on the tumor's oxygenation condition. A higher fraction of direct action generated by a high LET_d_ beam would eventually reduce the corresponding OER more effectively. The higher the LET_d_, the lower the OER is.^8^

In this study, we performed a proof‐of‐concept study to compare different LET_d_ modulation approaches for LAPC in order to deliver high RBE‐weighted doses by high LET_d_ carbon‐ion beams.

## 2 METHODS

### 2.1 Patient information

The patient's selection criterion was LAPC, and the tumor was located close to the stomach or duodenum. Ten patients were selected from our clinical database. They had been irradiated by CIRT with 67.5 Gy (RBE) in 15 fractions to the target. Their treatment plans were defined as the “original plan” thereafter.

### 2.2 CIRT technique

**FIGURE A1:**
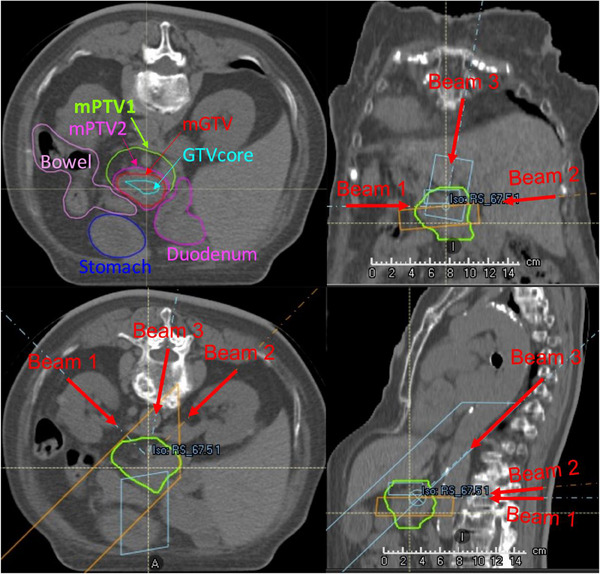
The schematic descriptions of modulated targets and the beam arrangements in a typical case.

Our center was facilitated with a Siemens synchrotron system (IONTRIS, Siemens, Germany), and only 45° and horizontal carbon‐ion beams are available. The patients were treated in the prone position, fixed with vacuum pads and back coverings. The patients were asked to fast for 2 h before the CT simulation. Anzai respiratory gating (Anzai medical cooperation, Japan) was used. Gating window was generally from 20% of exhale phase to 20% of inhale phase. Three 45° beams were arranged, two from the right and left lateral sides of the body, and one from the back through the spinal cord (Figure A1). The treatment plan was carried out on the average CT of gating window. The original plan was generated by Syngo (V13C, Siemens, Germany) with the local effect model I (LEM).^9^, ^10^ The dose was delivered by the pencil beam scanning technique^11^ with three ports daily.

### 2.3 The LET_d_ modulation approach

The treatment planning system: This dosimetric study was carried out by RayStation (V10B, RaySearch, Sweden). The image data from 10 patients were input into RayStation.

Definition of the modulated targets: Table 1 shows the definitions. The mPTV2 to GTVcore were the modulated targets, and their volumes became smaller and smaller gradually.

**TABLE 1:** Definition of the modulated targets.

Target name	Definition	Comment
GTV	The fused gross tumor volume in gating window respiratory phases	Gating window: from 20% of exhale to 20% of inhale
CTV	GTV+ uniformly 5 mm	
PTV1	CTV+ uniformly 5 mm	
PTV2	GTV+ uniformly 3 mm	
GI_PRV_	GI + uniformly 3 mm	
mPTV1	PTV1 − GI_PRV_	When GI_PRV_ was overlapped with PTV1
mPTV2	PTV2 − (GI_PRV_)	When GI_PRV_ was overlapped with PTV2
mGTV	GTV− (GI_PRV_)	When GTV was overlapped with GTV_PRV_
GTVcore	GTV uniformly shrinks 10 mm, or 5 to 10 mm when GTVcore was <2 mL	

Generating LET_d_ modulation plans: To increase the target LET_d_, we applied the following means: (1). More Bragg‐peak (BP) had put in the modulated targets; (2). To increase the BP density, higher doses were necessary; and (3). LET_d_ could be modulated to higher in small target volumes than in large target volumes; however, we had to keep dose constraints of the gastrointestinal (GI) tracts when high CIRT doses were delivered. Finally, we found that the simultaneous integrated boost technique (SIB) was an appropriate means to increase target LET_d_. We generated four SIB plans based on the re‐optimizations of the original plans using the same angles and primary target prescriptions: plan 1, plan 2, plan 3, and plan 4 (Table 2). Their SIB targets are mPTV2, mGTV, GTVcore, and GTVcore, respectively. The corresponding SIB prescriptions are listed in Table 2. The original plan was taken as the control plan to compare LET_d_ and related OER.

**TABLE 2:** The dose‐averaged linear energy transfer (LET_d_) modulation plans and their prescribed doses.

Plan name	Primary target	Primary prescription (Gy (RBE))	SIB target	SIB prescription (Gy (RBE))
Original plan	mPTV1	67.5	none	n/a
Plan 1	mPTV1	67.5	mPTV2	75.0
Plan 2	mPTV1	67.5	mGTV	75.0
Plan 3	mPTV1	67.5	GTVcore	75.0
Plan 4	mPTV1	67.5	GTVcore	100.0

*Note*: 100 Gy (RBE) is about the dose needed to eradicate the tumor hypoxia.

The target coverage requirements and GI constraints: ≥95% (D95%[Gy (RBE)]) of mPTV1 and all modulated targets should be covered by ≥95% of the prescribed doses; and GI constraints, including duodenum, stomach, and intestine were the same: the absolute volumes of GIs received doses ≥50 Gy (RBE) (V50) should be ≤2.0 mL, V32 ≤ 6.0 mL, and V21 ≤ 24.0 mL. For spinal cord, the maximum dose (D_max_) was ≤40.0 Gy (RBE), and for kidney, the percentage volume received 18 Gy (RBE) was <20%.

More investigations using a typical case: first, including plan 1 to plan 3, we generated plans using the modulated targets ranging from mPTV1 to GTVcore, but the prescribed doses to the modulated targets were the same as 75 Gy (RBE). The purpose was to study how the size of the modulated target impacts LET_d_. Second, including plan 3 and plan 4, we generated plans using GTVcore, but the prescribed dose to GTVcore increased from 67.5 to 100 Gy (RBE). We wanted to analyze how the prescribed dose to GTVcore impacts LET_d_.

### 2.4 LET_d_ and OER calculation

The CIRT LET_d_ in patients was calculated according to our approach.^12^ The conversion from LET_d_ to OER was based on a systematic study using CHO cells.^13^ Equation (1) is displayed as follows:

(1) $$\mathrm{OER}\;\left(pO_{2},\mathrm{LET}d\right)=\frac{b\left(Ma+\mathrm{LET}d^{\gamma }\right)/\left(\mathrm{LET}d^{\gamma }+a\right)+pO_{2}}{b+pO_{2}}\;$$

where *p*O_2_ is the tumor oxygen level. The oxygen concentration in LAPC was estimated at 0.5%, that is, 3 mmHg, based on the former study.^2^, ^14^ *b* = 0.25%,^15^ *a* = [8.27 ∙10^5^ (keV/ μm)]^γ^, and *γ* = 3.0. M = 2.7 being the maximum OER. When *p*O_2_ = 0.5% and LET_d_ > 48 keV/μm, OER < 1.5.

### 2.5 Statistical analysis

We first evaluated the RBE‐weighted dose coverage in GTV and GTVcore, that is, D95% as well as the dose received by GI tracts, that is, V50, V32, and V21, in all plans. Then, we evaluated the LET_d_s and OERs both in GTV and GTVcore. They were the LET_d_ and OER that 99% of the volume received, that is, LET_min_ and OER_max_, the mean LET_d_ (LET_mean_) and OER (OER_mean_). A study from Hagiwara et al.^16^ showed, to control LAPC, a minimum LET_d_ of 44 keV/μm was needed. Therefore, we also evaluated the percentage of volume receiving >LET_d_ of 44 keV/μm (V_44 keV/μm_ %). We also evaluate the minimum LET_d_ that 2 and 6 mL of GI organs received (LET_2 mL_ and LET_6 mL_) and the LET_mean_ that the liver and two kidneys received. The paired *t*‐tests were performed for LET_d_ and OER for different pairs of plans by SPSS (V24, IBM, USA). *p* value of <0.05 was considered as statistical significance.

## 3 RESULTS

Table 3 lists the RBE‐weighted dose, LET_d,_ and OER in GTV and GTVcore in original plan (the control plan) and four modulation plans. For GTV (Table 3), the D95% meets the requirements. Based on that, compared to that in original plan, LET_mean_ increases and OER_mean_ decreases in all modulating plans (all *p* < 0.05). As the modulated volumes decrease from plan 1 to plan 3, LET_mean_ remains similar. But, in plan 4 when the prescribed dose to GTVcore increases to 100 Gy (RBE), LET_mean_ increases significantly by 21.3% from 47.8 to 58.0 keV/μm (*p* < 0.05). OER_mean_ is lower in the modulation plans than that in original plan (all *p* < 0.05), and OER_mean_ in plan 4 is lowest (all *p* < 0.05). For the GTVcore where hypoxic cells resided (Table 4), compared to original plan, as the increase of D95%, the LET_mean_ increases from plan 1 to plan 4 (all *p* < 0.05). Most significantly in plan 4, LET_mean_ increases by 75.6%, from 51.3 keV/μm to 90.1 keV/μm (*p* < 0.05). OER_mean_ decreases slightly from plan 1 to plan 4, but in plan 4, they decrease more than the other plans. Compared to original plan, V_44 keV/μm_ increases in all modulation plans in both GTV and GTVcore (all *p* < 0.05). Especially in plan 4, it is 85% in GTV and 99.9% in GTVcore, whereas 63.9% and 50.1%, respectively in original plan (*p* < 0.05). The *p* values in statistical analyses of all LET_d_ and OER parameters for GTV and GTVcore are listed in Table A1. The OER_mean_ of GTVcore in plan 4 dropped to 1.41, which is smaller than the received doses (148% of the prescription). Therefore, the hypoxia may be eradicated in theory. Further improvement on MST is worth expecting.

**TABLE 3:** The relative biological effectiveness (RBE)‐weighted dose, dose‐averaged linear energy transfer (LET_d_)_,_ and oxygen enhancement ratio (OER) in gross tumor volume (GTV) for all plans.

Plan name	Modulated volume (mL)	D95% (Gy [RBE])	LET_min_ (keV/μm)	LET_mean_ (keV/μm)	V_44 keV/μm_(%)	OER_max_	OER_mean_
Original plan	n/a	66.54 (65.62–66.93)	36.5 (33.8–39.9)	47.8 (44.3–56.6)	63.9 (44.0–87.9)	1.54 (1.53–1.54)	1.50 (1.46–1.51)
Plan 1	51.7 (29.9–83.4)	73.84 (72.47–74.28)	37.8 (34.0–42.0)	49.5 (45.8–57.6)	73.5 (54.8–92.4)	1.53 (1.52–1.54)	1.49 (1.46–1.51)
Plan 2	29.6 (15.2–49.8)	73.05 (72.26–73.40)	37.0 (33.5–40.1)	50.1 (46.6–57.5)	74.8 (57.6–91.6)	1.53 (1.53–1.54)	1.49 (1.46–1.50)
Plan 3	2.5 (2.0–3.30)	66.50 (65.67–66.88)	34.7 (32.3–38.4)	49.1 (45.1–58.6)	69.8 (54.1–86.4)	1.54 (1.53–1.54)	1.49 (1.45–1.51)
Plan 4	2.5 (2.0–3.30)	65.19 (63.46–65.81)	34.6 (28.5–38.5)	58.0 (52.7–65.3)	85.0 (73.7–94.5)	1.54 (1.53–1.55)	1.45 (1.42–1.48)

**TABLE 4:** The relative biological effectiveness (RBE)‐weighted dose, dose‐averaged linear energy transfer (LET_d_)_,_ and oxygen enhancement ratio (OER) in gross tumor volume core (GTVcore) for all plans.

Plan name	Modulated volume (mL)	D95% (Gy [RBE])	LET_min_ (keV/μm)	LET_mean_ (keV/μm)	V_44 keV/μm_ (%)	OER_max_	OER_mean_
Original plan	n/a	66.74 (66.35–66.90)	38.6 (36.2–41.8)	51.3 (46.5–61.3)	50.1 (10.1–85.8)	1.53 (1.52–1.54)	1.51 (1.50–1.52)
Plan 1	51.7 (29.9–83.4)	74.10 (73.59–74.38)	39.7 (35.4–43.5)	52.8 (47.8–61.2	62.6 (16.7–94.5)	1.53 (1.52–1.54)	1.51 (1.49–1.52)
Plan 2	29.6 (15.2–49.8)	74.17 (73.75–74.36)	39.6 (36.1–43.7)	53.2 (48.7–60.1)	65.1 (25.5–96.6)	1.53 (1.51–1.54)	1.51 (1.50–1.52)
Plan 3	2.5 (2.0–3.30)	73.83 (73.54–74.13)	41.8 (37.4–44.7)	59.8 (52.9–70.3)	88.7 (56.5–99.4)	1.52 (1.51–1.53)	1.49 (1.47–1.51)
Plan 4	2.5 (2.0–3.30)	93.74 (92.47–95.13)	51.2 (44.6–55.3)	90.1 (77.4–99.6)	99.9 (99.7–100)	1.49 (1.47–1.51)	1.41 (1.38–1.45)

The RBE‐weighted dose volumes received by GI for all plans are listed in Table 5, which meets the requirements in all cases for all plans. The LET_2 mL_ and LET_6 mL_ in the bowel, duodenum, and stomach, and the LET_mean_ in the liver and two kidneys for all the plans are shown in Table 6, and *p* values in statistical analyses are in Table A2. LET_2 mL_ and LET_6 mL_ in GIs and LET_mean_ in liver and kidneys were similar among all plans, except for plan 4, where the values are lower than the other plans.

**TABLE 5:** The relative biological effectiveness (RBE)‐weighted dose volumes received by gastrointestinal (GI) tracts for all plans.

Plan name	Bowel			Duodenum			Stomach		
	V50 < 2 mL	V32 < 6 mL	V21 < 24 mL	V50 < 2 mL	V32 < 6 mL	V21 < 24 mL	V50 < 2 mL	V32 < 6 mL	V21 < 24 mL
Original plan	0.48 (0.0–1.34)	2.51 (0.35–5.79)	6.40 (1.78–20.80)	0.21 (0.0–0.96)	1.57 (0.0‐5.32)	4.12 (0.11–12.64)	0.20 (0.0–0.43)	2.44 (0.0–5.69)	8.32 (1.17–17.78)
Plan 1	0.55 (0.0–1.79)	2.54 (0.31–5.94)	6.60 (1.96–21.15)	0.29 (0.0–1.08)	1.57 (0.0–5.42)	4.12 (0.08–12.39)	0.24 (0.0–0.66)	2.33 (0.01–5.39)	8.26 (1.12–18.16)
Plan 2	0.45 (0.0–1.24)	2.41 (0.25–5.71)	6.18 (1.59–20.17)	0.29 (0.0–1.03)	1.62 (0.0–5.69)	4.28 (0.09–13.01)	0.23 (0.0–0.70)	2.36 (0.01–5.70)	8.21 (1.01–17.02)
Plan 3	0.43 (0.0–1.19)	2.37 (0.20–5.50)	6.08 (1.31–20.51)	0.20 (0.0–0.77)	1.4 9(0.0–5.58)	4.06 (0.08–13.95)	0.19 (0.0–0.44)	2.28 (0.0–5.75)	7.64 (0.93–18.27)
Plan 4	0.39 (0.0–1.29)	2.18 (0.21–4.82)	5.37 (1.08–14.48)	0.16 (0.0–0.56)	1.69 (0.0–5.76)	4.79 (0.09–15.21)	0.17 (0.0–0.39)	2.16 (0.0–4.85)	7.93 (0.95–18.25)

**TABLE 6:** Dose‐averaged linear energy transfer (LET_d_) parameters in the adjacent organ at risk (OARs) for all plans.

	Bowel		Duodenum		Stomach		Liver	Kidney‐L	Kidney‐R
Plan name	LET_2 mL_ (keV/μm)	LET_6 mL_ (keV/μm)	LET_2 mL_ (keV/μm)	LET_mean (ke/μm)_	LET_2 mL_ (keV/μm)	LET_6 mL_ (keV/μm)	LET_mean_ (keV/μm)	LET_mean_ (keV/μm)	LET_mean_ (keV/μm)
Original plan	71.1 (49.7–99.9)	54.8 (39.2–86.9)	63.8 (46.0–88.4)	51.1 (39.4–71.0)	86.3 (53.0–102.8)	70.7 (47.7–85.0)	11.7 (6.9–17.2)	12.1 (8.1–16.4)	9.4 (6.4–13.1)
Plan 1	71.6 (48.8–100.7)	54.2 (38.8–86.8)	64.5 (46.7–92.6)	50.7 (40.3–68.3)	84.5 (52.3–102.0)	69.1 (47.5–84.1)	12.3 (7.2–19.2)	14.1 (8.1–29.5)	10.4 (6.8–18.5)
Plan 2	71.7 (47.7–102.0)	53.9 (36.4–87.9)	64.4 (46.9–91.8)	51.2 (40.2–70.8)	84.8 (53.1–100.7)	69.5 (47.6–83.5)	11.6 (6.8–17.0)	11.9 (7.8–16.3)	9.4 (6.6–12.8)
Plan 3	72.3 (49.6–103.3)	54.8 (37.5–89.5)	64.7 (46.6–91.5)	51.7 (39.2–71.1)	85.4 (54.3–101.4)	70.2 (48.5–84.3)	11.3 (6.6–16.7)	11.8 (7.7–15.8)	9.3 (6.4–12.9)
Plan 4	68.4 (50.0–100.0)	51.5 (35.2–81.8)	61.8 (47.3–91.2)	50.8 (37.8–70.4)	82.6 (56.8–96.0)	67.2 (44.5–79.2)	11.0 (6.6–11.7)	11.5 (7.6–15.3)	9.1 (6.2–12.7)

**FIGURE 1:**
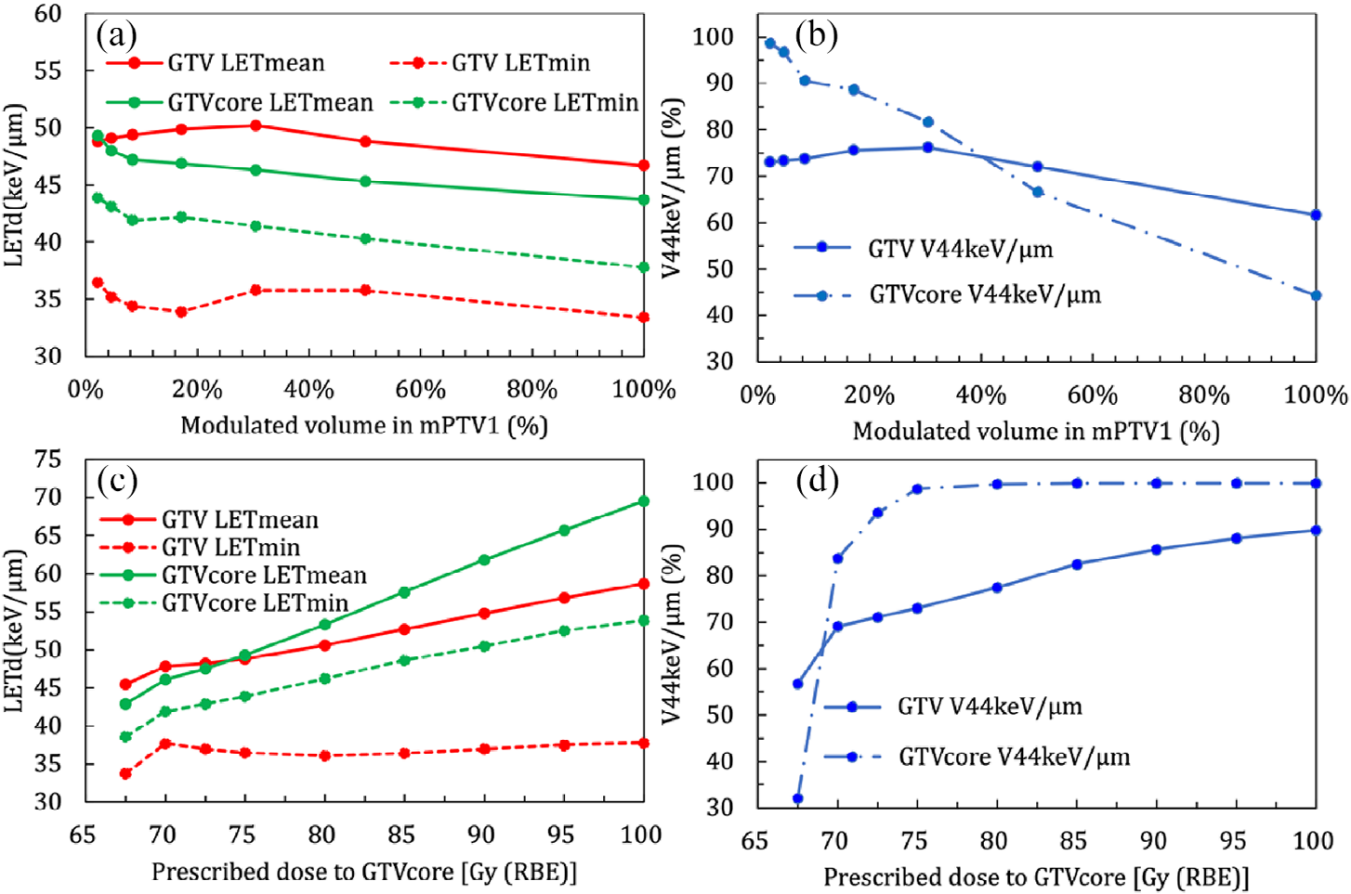
The dose‐averaged linear energy transfer (LET_d_) changes as the modulated volumes and prescribed dose change in a typical case. (a) and (b): the modulated volumes change from mPTV1 to gross tumor volume core (GTVcore) while using the simultaneous integrated boost technique (SIB) dose as 75 Gy (RBE) relative biological effectiveness; (c) and (d): the prescribed doses to GTVcore increase from 67.5 to 100 Gy (RBE). Red solid and dash lines: GTV LET_mean_ and GTV LET_min_; green solid and dash lines: GTVcore LET_mean_ and GTVcore LET_min_; blue solid and dash lines: GTV V_44 keV/μm_ and GTVcore V_44 keV/μm._

**FIGURE 2:**
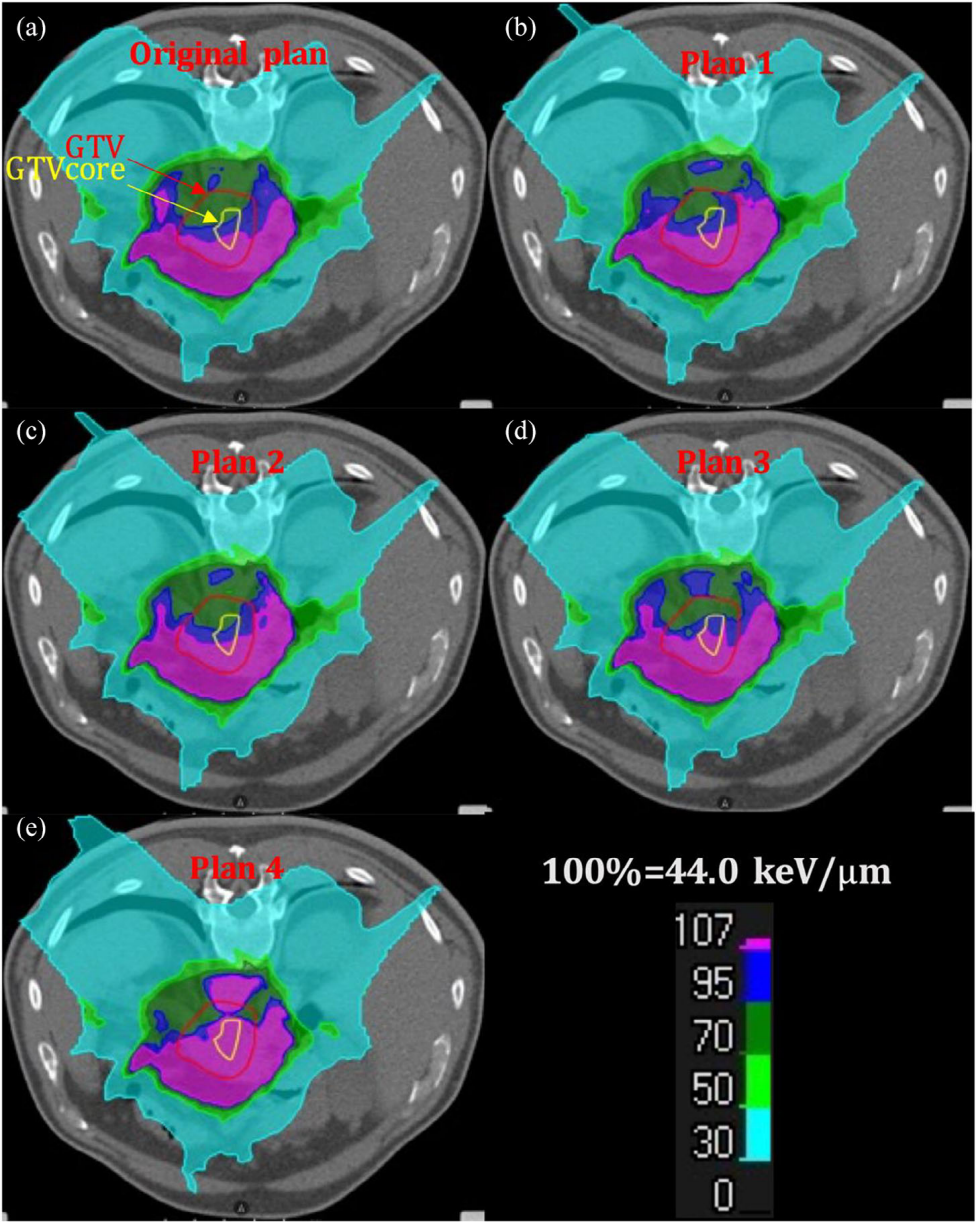
The dose‐averaged linear energy transfer (LET_d_) distributions in a typical case. (a): original plan; (b): plan 1; (c): plan 2; (d): plan 3; and (e): plan 4. The areas circled by red and yellow lines are the gross tumor volume (GTV) and gross tumor volume core (GTVcore), respectively.

**FIGURE A2:**
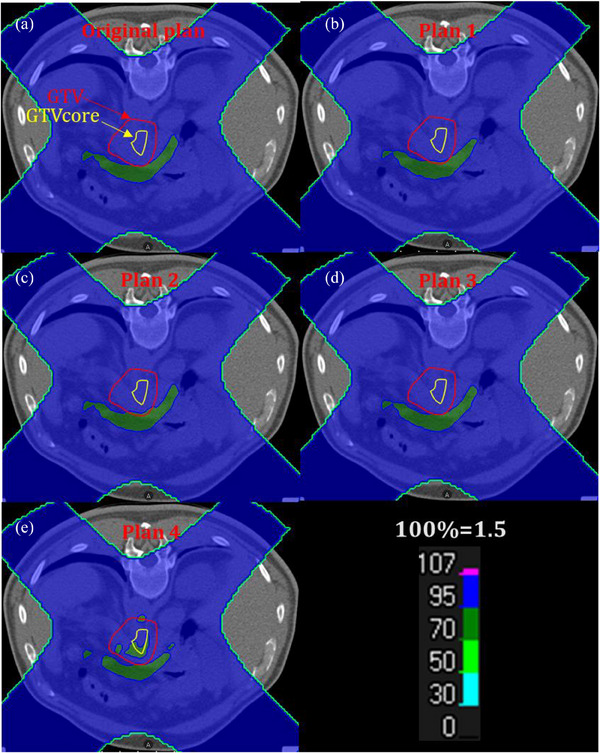
The oxygen enhancement ratio (OER) distributions: original plan (a), plan 1 (b), plan 2 (c), plan 3 (d) plan 4 (e). The red line was gross tumor volume (GTV). The blue area represents OER of 1.4, and the dark green area, OER of 1.0.

Figure 1 shows how the size of the modulated targets and the corresponding prescribed doses affect LET_d_ based on a typical case. Based on the same SIB dose, as the modulated volumes increase, the LET_mean_, LET_min_, and V_44 keV/μm_ in GTV and GTVcore tend to decrease (Figure 1a,b). Furthermore, if the modulated volumes have similar sizes and locations to GTVcore, the GTVcore LET_d_s can improve effectively. When the prescribed dose to GTVcore increases from 67.5 to 100 Gy (RBE), the LET_mean_, LET_min_, and V_44 keV/μm_ in both GTV and GTVcore increase (Figure 1c,d). Figure 2 shows the LET_d_ distributions of original plan (a), plan 1 (b), plan 2 (c), plan 3 (d), and plan 4 (e) in this case. The high LET_d_ region, that is, >44.0 keV/μm, is located at the beam distal part in the target, and mostly outside of GTV. Only in plan 4, high LET_d_ covers most of GTV and the entire GTVcore. The high LET_d_ extending to the spinal cord is mainly caused by the LET_d_ overlap from three beams. The safety of the spinal cord can be maintained the same as the original plan since this part is located at the proximal of all beams and is still far away from the spinal cord. Figure A2 shows the corresponding OER distributions. Similar to LET_d_ distribution, the volume with the lowest OER of 1.0 is located at the high LET_d_ volume (beam distal) and is mostly outside of GTV. Only in plan 4, the OER of 1.0 is within GTVcore.

## 4 DISCUSSION

Monlinelli et al reported 50 sacral chordomas treated by CIRT and found local recurrences at the high‐dose regions in 13 patients. Further analysis showed CTVs received significantly lower median LET_d_ compared to patients without local recurrences (27.4 vs. 30.2 keV/μm, *p* = 0.000).^7^ This study shows that based on the same treatment protocol, patients who received lower LET_d_ are more likely to recurrent. Matsumoto et al reported 30 chondrosarcomas treated by CIRT, tumor‐controlled in 19 patients, and local failure in 11 patients. The LET_d_ in PTV was lower in the local failure patients than that in local control patients (37.8–46.8 vs. 38.0–70.6 keV/μm).^17^ The percentage of volume irradiated with <50 keV/μm in total tumor volume was smaller in local failure patients than that in the patients with tumor control. This study however does not present a LET_d_ cut‐off value between local control and local recurrence group. It does show patients whose PTVs receive lower LET_d_ are likely to recurrent. Both studies verified that the CIRT LET_d_ was also critical for tumor eradication. It was known that the same absorbed CIRT dose could be deposited by different energies of carbon‐ion beams with different LET_d_. Higher LET_d_ yielded stronger biological effects. Therefore, we may need to deliver high enough RBE‐weighted doses by high LET_d_ carbon‐ion beams. Another advantage of using high LET_d_ beams was to decrease OER.^8^ This was especially meaningful for LAPC, a hypoxic carcinoma. Therefore, we tried to deliver high doses to the tumor by high LET_d_ carbon‐ion beam as high as possible in this study.

The dose within GTVcore in the original plan was delivered by the multiple plateau doses in front of BP, which were low LET beams. Therefore, the high LET in GTVcore was diluted. For this reason, we have to put more BP in GTVcore to increase LET_d_. While, in plan 1 to plan 4, by using SIB, the locally increased dose was mainly deposited by BP, which had the highest LET_d_. With increasing of SIB dose meanwhile decreasing the boost volume, the density of BP would significantly increase, thus raising the LET_d_. As CIRT yielded a direct effect on DNA with 60%−70% of DNA injury being DSB, its radiation injury does not rely on oxygen. Experiments in vitro and in vivo demonstrated that OER in CIRT was reduced to around 2, and the higher carbon‐ion LET, the lower OER.^8^

We used three 45° beams at the patient's prone position. The advantage of this beam arrangement was that the beams went through relatively homogenous tissues and avoided gas in GI so as to decrease beam range uncertainties. However, this plan had two disadvantages: (1). GIs were located at the distal of the beams, where the beam LET_d_ peaked; and (2). this beam arrangement also may bring high doses in GIs. To ensure safety, after 1 cm contraction based on GTV, if the GTVcore is >2 mL, it would be the modulated volume. If not, the contraction would reduce to 0.5 cm in some directions but maintain 1 cm away from GI all the time. Second, the beam from the spinal cord has a very long range. Therefore, the dose loading of the spine beam cannot be too high. Third, all beams should avoid going through the lung tissue. For one thing, the accuracy of the pencil beam algorithm in the lung is not satisfied. For another thing, the movement of the diaphragm creates range uncertainty and interplay effect. Lastly, the air cavity in GI should also be avoided. Therefore, we asked the patient to keep a similar daily diet and avoid food that could produce gas in the GI. Besides, we do CT simulation and give irradiation after patient fast for at least 2 h.

For LAPC to increase the CIRT dose uniformly was difficult due to the limits of GI constraints. Whereas, increasing doses to part of the tumor was possible. In phantom studies, LET_d_ strongly depended on the target volume, that is, quickly increasing with decreasing volumes.^18^ Thus, decreasing modulated volume may increase LET_d_, and also spare GI. We used SIB to simultaneously deliver a basic dose and a high boost dose. The modulation parameters were the volume, the location of targets within GTV, and the corresponding prescribed doses to modulated volumes. In original plan, 67.5 Gy (RBE) was delivered uniformly to mPTV1. When the prescribed dose to the modulated volumes was increased to 75 Gy (RBE), LET_d_ increased in GTV and GTVcore for all modulation plans referring to original plan. As the modulated volumes were shrunk from plan 1 (51.7 mL) to plan 3 (2.5 mL), LET_d_ always increased in GTVcore. When the prescribed dose to GTVcore was further increased to 100 Gy (RBE), LET_d_ could increase significantly (Tables 3 and 4 and Figure 2e). Moreover, as reported by Hagiwara,^16^ to control LAPC, a minimum LET_d_ of 44 keV/μm was needed. V_44 keV/μm_ in GTVcore increased from original plan (50.1%) to 88.7% in plan 3, but unfortunately, only 69.8% of GTV received doses from >44 keV/μm (Figure 2). To further move the high LET_d_ region to GTV, we increased the prescribed dose to 100 Gy (RBE) in plan 4. V_44 keV/μm_ of GTVcore and GTV were increased to 99.9% and 85.0%. The study based on the typical case reaffirmed the aforementioned discovery. As for the LET_d_ to OARs, the LET_2 mL_ and LET_6 mL_ in GIs and LET_mean_ in liver and kidneys in plan 4 were also lower than the other plans.

To deliver a high CIRT dose to target by high LET_d_ beams, we believe that SIB is a good way to achieve this goal. Our study showed that all modulation plans could meet the goal, but plan 4 works better. However, how to locate the boost target was another issue. We thought that ^[18F]^FMISO‐PET/CT would be of great help in identifying the location of hypoxic cells.^19^ Once the hypoxic area is identified we will fuse FMISO‐PET images with the simulation CTs to design the dose boost volume.

To effectively eradicate the radio‐resistant LAPC, high doses by high LET_d_ beam are necessary. The better approach to increase beam LET_d_ is the use of heavier particles, like neon and oxygen ions.^20^, ^21^ However, those heavy ion beams are not available for clinical application currently. In our center, we have already initiated a prospective clinical trial for LAPC (NCT05424159). In this trial, we modified GTV by leaving a gap of 3 mm between GTV and GI to avoid a high dose within GI. We think that the hypoxic cells are located in the core of the tumor, and the peripheral part of the tumor would be less hypoxia. We use the SIB technique to deliver a higher dose (75.0 Gy (RBE)) to the core of the tumor, meanwhile keeping GI within the tolerance doses. We hope and anticipate that 75.0 Gy (RBE) will result in higher local control than before without severe complications.

More studies are still needed. First, due to the range uncertainty, SIB could potentially lead to an overshoot in OARs. Thus, we may need further research to evaluate and improve the plan's robustness. Secondly, based on photon or proton experience, increasing the dose itself can improve the outcome. If it really improves, we need to analyze which factor plays the major role, that is, dose or LET_d_. Thirdly, as we see in the original plan of a typical case, the LET_d_ even in the GTVcore shows a notable gradient. It implies that a uniform dose boost may not be necessary. The voxel‐based LET_d_ analysis and the dose boosting based on the analysis may be preferred.

Not all LAPCs may be suitable for this technique. Patients with large tumor size may be more beneficial since a larger target leads to lower LET_d_ at the center, Low LET_d_ patients are more likely to recurrent. Secondly, our clinical experience shows these patients who perform well in setup repeatability, motion management, and treatment preparation are more suitable for SIB treatment. We also believe that besides LAPC, the CIRT SIB technique could be tried for those tumors with a high percentage of hypoxic cells, like big volumes of non‐small cell lung cancers, big hepatocellular carcinomas, and soft tissue sarcomas.

## 5 CONCLUSIONS

For CIRT in LAPC, we have to pay attention to not only the prescribed dose but also the beam LET_d_. Higher LET_d_ carbon‐ion beams yield higher RBE and lower OER. In our original plan, we could deliver 67.5 Gy (RBE), but the beam LET_d_ was probably not high enough to kill hypoxic cells. By SIB to up‐modulate LET_d_ in the targets, we could deliver doses of 75 to 100 Gy (RBE) to the tumor core, meantime the high dose boost focusing on a very localized volume within GTV would help to increase the GTV LET_d_, thus reducing the corresponding OER.

## AUTHOR CONTRIBUTIONS

Guo‐Liang Jiang and Zheng Wang designed the study. Weiwei Wang performed the study. Jiayao Sun, Jingfang Zhao, and Jingyi Cheng helped analyzed the results. Weiwei Wang, Guo‐Liang Jiang, and Zheng Wang wrote the manuscript. All authors reviewed and approved the manuscript.

## CONFLICT OF INTEREST STATEMENT

The authors declare no conflict of interest.

## Appendix

See Figures A1, A2 and Tables A1, A2

**TABLE A1:** *p* Values of statistics between pair of plans in gross tumor volume (GTV) and gross tumor volume core (GTVcore).

GTV						
Plan name	D95%	LET_min_	LET_mean_	V_44 keV/μm_	OER_max_	OER_mean_
OP versus P1	***0.000***	***0.055***	***0.000***	***0.004***	0.102	***0.008***
OP versus P2	***0.000***	0.182	***0.000***	***0.000***	0.317	***0.003***
OP versus P3	*0.583*	***0.002***	***0.000***	***0.010***	***0.046***	***0.014***
OP versus P4	***0.000***	***0.050***	***0.000***	***0.000***	0.083	***0.004***
P1 versus P2	***0.000***	0.089	***0.021***	0.447	0.083	**0.157**
P1 versus P3	***0.000***	***0.002***	**0.177**	0.135	***0.023***	0.564
P1 versus P4	***0.000***	***0.021***	***0.000***	***0.018***	***0.038***	***0.005***
P2 versus P3	***0.000***	***0.000***	***0.007***	***0.008***	***0.025***	0.180
P2 versus P4	***0.000***	***0.023***	***0.000***	***0.008***	***0.046***	***0.005***
P3 versus P4	***0.000***	0.940	***0.000***	***0.000***	***0.007***	***0.004***
GTVcore						
OP versus P1	***0.000***	***0.053***	***0.035***	***0.022***	***0.009***	0.096
OP versus P2	***0.000***	***0.021***	***0.040***	***0.028***	***0.013***	***0.046***
OP versus P3	***0.000***	***0.010***	***0.000***	***0.005***	***0.004***	***0.005***
OP versus P4	***0.000***	***0.000***	***0.000***	***0.005***	***0.005***	***0.005***
P1 versus P2	*0.122*	0.670	0.747	0.646	***0.180***	0.655
P1 versus P3	***0.004***	***0.070***	***0.000***	***0.005***	***0.004***	***0.004***
P1versus P4	***0.000***	***0.000***	***0.000***	***0.005***	***0.005***	***0.005***
P2 versus P3	***0.001***	***0.040***	***0.000***	***0.007***	***0.005***	***0.004***
P2 versus P4	***0.000***	***0.000***	***0.000***	***0.005***	***0.004***	***0.005***
P3 versus P4	***0.000***	***0.000***	***0.000***	***0.005***	***0.005***	***0.005***

Abbreviations: LET, linear energy transfer; OP, original plan; OER, oxygen enhancement ratio; P1, plan 1;P2, plan 2;P3, plan 3;P4, plan 4.

**TABLE A2:** *p* Values of statistics between pair of plans in GIs, liver, and kidneys.

Plan name	Bowel		Duodenum		Stomach		Liver	Kidney‐L	Kidney‐R
	LET_2 mL_	LET_6 mL_	LET_2 mL_	LET_6 mL_	LET_2 mL_	LET_6 mL_	LET_mean_	LET_mean_	LET_mean_
OP versus P1	*0.538*	*0.215*	*0.538*	*0.646*	***0.002***	***0.002***	*0.344*	*0.316*	*0.355*
OP versus P2	*0.414*	*0.095*	*0.447*	*0.721*	***0.009***	***0.030***	***0.041***	*0.275*	*1.000*
OP versus P3	*0.163*	*0.931*	*0.202*	*0.240*	*0.089*	*0.328*	***0.000***	***0.017***	*0.147*
OP versus P4	***0.034***	***0.000***	*0.294*	*0.444*	***0.029***	***0.002***	***0.000***	***0.005***	*0.108*
P1 versus P2	*0.807*	*0.367*	*0.855*	*0.332*	*0.497*	*0.196*	*0.287*	*0.289*	*0.344*
P1 versus P3	*0.255*	*0.303*	*0.815*	*0.153*	*0.136*	***0.002***	*0.148*	*0.248*	*0.307*
P1 versus P4	***0.013***	***0.003***	*0.131*	*0.959*	*0.220*	*0.054*	*0.073*	*0.212*	*0.250*
P2 versus P3	*0.160*	*0.086*	*0.535*	*0.046*	*0.220*	***0.001***	***0.000***	***0.033***	*0.134*
P2 versus P4	***0.006***	***0.005***	*0.111*	*0.359*	*0.073*	***0.006***	***0.000***	***0.006***	***0.003***
P3 versus P4	***0.001***	***0.000***	***0.042***	***0.022***	***0.049***	***0.003***	***0.001***	***0.031***	*0.147*

Abbreviations: LET, linear energy transfer; OP, original plan; P1, plan 1;P2, plan 2;P3, plan 3;P4, plan 4.
